# The Economic Burden Associated with Depressive Symptoms among Middle-Aged and Elderly People with Chronic Diseases in China

**DOI:** 10.3390/ijerph191912958

**Published:** 2022-10-10

**Authors:** Yun Wu, Sihui Jin, Jianwei Guo, Yi Zhu, Lijin Chen, Yixiang Huang

**Affiliations:** 1 Department of Health Policy and Management, School of Public Health, Sun Yat-sen University, 74, Zhongshan 2nd Road, Guangzhou 510030, China; 2 Department of Epidemiology and Health Statistics, School of Public Health, Huazhong University of Science and Technology, 1037 Luoyu Road, Hongshan District, Wuhan 430074, China

**Keywords:** economic burden, depressive symptoms, chronic disease, multimorbidity

## Abstract

Coexisting physical diseases and depressive symptoms exacerbate morbidity and disability, but their incremental economic burden remains unclear. We used cross-sectional data from the China Health and Retirement Longitudinal Study (CHARLS) survey in 2018 to estimate the economic burden associated with depressive symptoms among middle-aged and elderly people with chronic diseases. A multivariable regression model was used to assess the annual health care utilization, expenditures, and productivity loss of depressive symptoms among people with 12 common chronic diseases. We found that depressive symptoms were associated with higher incremental economic burdens, as the total health care costs increased by 3.1% to 85.0% and annual productivity loss increased by 1.6% to 90.1%. Those with cancer or malignant tumors had the largest economic burden associated with depressive symptoms, with CNY 17,273.7 additional annual health care costs and a loss of CNY 2196.2 due to additional annual productivity loss. The effect of depressive symptoms on the economic burden of patients with chronic conditions did not increase by the number of chronic conditions. Considering the high economic burden associated with depressive symptoms among patients with chronic conditions, it is important to consider the mental health of patients in chronic disease treatment and management.

## 1. Introduction

Depression is a leading cause of disability worldwide and contributes greatly to the global disease burden. According to an extensive epidemiological survey, the lifetime prevalence of depression ranged from 10% to 15%, affecting more than 350 million people globally [1,2]. Studies have shown a higher prevalence of depression in patients with chronic conditions, including patients with cardiovascular disease (17–27%) [3], diabetes (11–31%) [4], and arthritis (10–24%) [5]. The World Health Organization Health Survey also found that people with at least one chronic disease (9.3–23%) had a higher prevalence of depression than those without chronic disease (3.2%) [6]. Depressive symptoms are the main clinical features of depression and a person can be diagnosed with depression if he/she shows a persistent and high level of depressive symptoms [7]. In China, the prevalence of depressive symptoms among elderly people with chronic diseases was 40.3% in 2018, especially in rural areas (72%) [8]. In addition, coexisting with more chronic conditions was associated with a higher risk of depressive symptoms. The presence of two or more chronic diseases was commonly defined as multimorbidity [9]. Patients with multimorbidity had twice the risk of depressive symptoms compared with those without multimorbidity, and the odds of having depressive symptoms was 45% higher for each additional chronic disease, compared with those without chronic disease [10].

The relationship between chronic disease and depression can be bidirectional. On the one hand, chronic conditions may contribute to the occurrence of depressive symptoms through factors such as disability, decreased quality of life and chronic physical pain [11,12]. On the other hand, depression may contribute to the development of chronic diseases by affecting the mediators of functional health, including limited social activity, sleep disorders, and unhealthy behaviors [13,14]. Additionally, many studies have shown that depressive symptoms are associated with multiple adverse outcomes for patients with chronic diseases, such as reduced treatment adherence and higher mortality [15,16].

Co-occurring depressive symptoms can lead to higher health care costs for patients with chronic diseases through increased hospitalizations, emergency department visits, and length of hospital stay [17,18]. A study of chronic obstructive pulmonary disease (COPD) patients in the United States showed that those with depression were 77% more likely to be hospitalized and 48% more likely to visit the emergency room, compared to COPD patients without depression [19]. Except for the impact on health care utilization, co-occurring depression can adversely impact workplace productivity. Compared with non-depressed patients, patients with depressive symptoms were more likely to have days off work (absenteeism) and be less productive at work (presenteeism) [20].

Given that the prevalence rate of coexisting chronic diseases and depressive symptoms is increasing, additional attention to this relationship is necessary. Many previous research focused on the effects of somatic functions and treatment caused by depressive symptoms among people with chronic conditions, but limited on economic burden. Our study aimed to assess and compare the economic burden of depressive symptoms among patients with 12 common chronic diseases. Additionally, we examined whether the economic burden rose with the number of chronic diseases. Assessing the impacts of depressive symptoms on chronic patients’ economic burden is significant. It will add value to the health policy-making process for the government and promote health care delivery models for primary health institutions.

## 2. Materials and Methods

### 2.1. Data Source

Our study’s data were from the 2018 China Health and Retirement Longitudinal Study (CHARLS), an ongoing nationally representative cohort study of Chinese adults aged 45 and over and their family members. The CHARLS baseline survey was conducted in 2011, covering 450 villages and urban communities from 30 provinces, and it was conducted every 2 years. The CHARLS was approved by the Ethical Review Committee of Peking University, and detailed descriptions of the survey design and procedures were reported in the original study documentation [21]. The CHARLS questionnaire collected information on general social statistical characteristics, health status and function, health care and insurance, work, retirement and pension, income and expenditure.

### 2.2. Study Sample

The subject population of our study was people aged 45 years or older with chronic diseases in China. Chronic diseases were diagnosed according to the question “Have you been diagnosed with the following conditions by a doctor?” The conditions were the 12 common chronic diseases included in our study, covering hypertension, dyslipidemia, diabetes, cancer or malignant tumor, chronic lung disease, liver disease, heart attack, stroke, kidney disease, stomach or other digestive diseases, arthritis or rheumatism and asthma. For hypertension, diabetes and chronic lung disease, in addition to the patients diagnosed by doctors, we also included self-reported patients and those identified by CHARLS physical examination.

A total of 19,816 individuals participated in the 2018 CHARLS survey. The inclusion criteria for this study were people aged ≥ 45, suffering from at least one chronic disease (the 12 common chronic diseases listed above) and completed information about sociodemographics, chronic disease, depressive states and health care expenditure. Finally, 12,458 respondents fulfilled the inclusion criteria and were included in this study.

### 2.3. Criteria for Depressive Symptoms 

Depressive symptoms were measured using the Center for Epidemiologic Studies Depression Scale (CES-D-10), which has been validated with elderly respondents in China [22]. The response scale for the CES-D-10 contains two positive questions and eight negative questions and each question has four response options, including rarely or none of the time, some, occasionally, and most or all of the time. The score of the 4 options ranges from 0 to 3, with a total score ranging from 0 to 30 and with a higher score indicating more serious depressive symptoms. Consistent with other studies, we used a cut-off score of ≥10 to distinguish participants with significant depressive symptoms [23,24].

### 2.4. Health Care Costs 

This study used the annual health care costs to represent the direct economic burden of disease, including utilization and expenditure of outpatient visit, hospitalization and self-medication. In addition, the self-medication cost was defined as the expenditure for treatment without resorting to professional medical care, including over-the-counter drugs, traditional herbs or medication, tonic/health supplements, and the use of health care equipment [25]. CHARLS asks respondents about the visits and costs of outpatient and self-medication in the past month, and the visits and costs of hospitalizations in the past year. Therefore, only annual hospitalization costs data were directly available, and the annual visit and cost of outpatients and self-medication were calculated by multiplying the amount in the most recent month by 12 [26,27]. All costs were presented in Chinese Yuan (CNY) and the official conversion rate was CNY 6.6 per USD 1.0 in 2018. 

### 2.5. Productivity Loss 

Our study estimated productivity loss using the human capital method to represent the indirect economic burden of people with chronic diseases. The productivity loss was estimated by assessing the value of missed days of work (absenteeism) and the days the participants were unable to work due to illness or disability (unemployment) [28]. After considering the wage difference between rural and urban residents, we divided the productivity loss into the following three categories: unemployment loss, agricultural loss and employed loss. CHARLS surveyed the number of missed days and unemployment in the past year due to health problems. Thus, the economic agricultural and employed losses were calculated by multiplying the average daily wage (CNY 104.8 for urban residents/CNY 59.3 for rural residents) with the number of missed days in a year. Unemployment cost was calculated by multiplying the average monthly wage (CNY 2183.8 for urban residents/CNY 1234.3 for rural residents) with the number of unemployment months in a year [29]. The average monthly/daily wage incomes were based on the per capital wage income of (urban/rural) residents published by the China National Bureau of Statistics in 2012, adjusted to 2018 levels by the Gross Domestic Product index [30].

### 2.6. Variables

The key explanatory variable of our study was depressive symptoms. Dependent variables in our study were direct and indirect economic burden, including health care visits and costs, missed work days and corresponding productivity loss. For selecting other independent variables, we used Anderson’s behavior model from the perspective of the influence on health care utilization, including predisposing factors, enabling factors, and need factors [31]. We included gender, age and marital status as predisposing factors. Enabling factors included residence district (categorized into rural and urban), education level, occupation, medical insurance types, and monthly household expenditure (averaged into 4 levels). Need factors consisted of perceived health status (categorized into good, fair and poor) and number of chronic diseases (categorized into 1, 2, 3, 4 and above) [32,33,34].

### 2.7. Statistical Analysis

We adopted mean/median imputation to fill the missing values of the key continuous variables after checking the distributions. The chi-square test was used to compare the demographic differences between patients with depressive symptoms and without depressive symptoms. For multivariate analysis, we used zero-inflated models because there was a large number of zero values both in health care visits and missed work days. A zero-inflated negative binomial regression model was applied to analyze outpatient visits, self-medication visits and missed work days, due to the discrete nature of the data. To analyze hospitalization visits, we used a zero-inflated Poisson regression model [35]. To overcome the common challenges related to modeling health care costs and productivity loss (e.g., high positive skewness and heteroscedasticity), we applied a generalized linear model (GLM) with gamma distribution and a log link for health care costs [34]. In addition, GLM with negative binomial distribution and a log link were used to estimate the economic loss of productivity [36]. We calculated the marginal effect of depressive symptoms on each outcome. We adjusted for gender, age, marital status, residence district, education level, health and comorbidity status in all models. A *p*-value of ≤0.05 was considered statistically significant.

## 3. Results

### 3.1. Prevalence of Depressive Symptoms and Demographic Characteristics

The prevalence of depressive symptoms among middle-aged and elderly patients with chronic diseases was 39.8%. Among the twelve common chronic diseases, the top three prevalence rates of depressive symptoms were asthma (51.9%), kidney disease (49.4%) and stroke (48.7%). In patients with multiple chronic conditions, the prevalence rate of depressive symptom onset in patients with four or more chronic diseases was the highest, at 52.5% (Table A1). 

A greater proportion of the people with chronic diseases that had depressive symptoms were female (31.8% vs. 47.1%; *p* < 0.001), separated/divorced/unmarried (51.4% vs. 38.2%; *p* < 0.001), rural residents (43.5% vs. 27.5%; *p* < 0.001), with no formal education (49.0% vs. 24.0%; *p* < 0.001), unemployed (44.1% vs. 25.8%; *p* < 0.001), with no insurance (50.0% vs. 23.0%; *p* < 0.001), with lower household monthly expenditure (48.7% vs. 31.5%; *p* < 0.001), poor health status (62.0% vs. 20.4%; *p* < 0.001) and with four or more chronic disease conditions (52.5% vs. 31.0%; *p* < 0.001) (Table 1).

### 3.2. Health Care Cost Estimation of Depressive Symptoms

**Figure 1:**
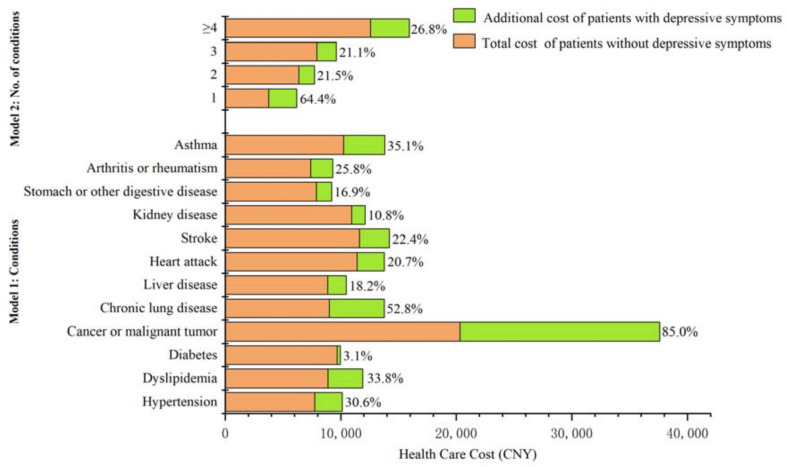
Comparing the proportion of additional health care costs to the total costs of patients without depressive symptoms. Note: The data details used in drawing Figure 1 are shown in Table A2.

The results showed that depressive symptoms significantly impact the use and cost of health care among middle-aged and elderly people with chronic diseases. In the 12 types of chronic disease groups, the additional health care costs of depressive symptoms accounted for between 3.1% and 85.0% of total health care costs of patients without depressive symptoms (Figure 1). The strongest effect was observed in patients with cancer or malignant tumors, which reached CNY 17,273.7 for 2.6 additional outpatient visits, 0.5 additional hospitalization visits and 2.3 additional self-medication visits (*p* < 0.05 for both), followed by chronic lung disease and asthma and the additional annual health care costs were CNY 4759.4 and CNY 3589.0, respectively (*p* < 0.05 for both). In terms of health care use, having depressive symptoms was significantly associated with increased outpatient visits but not hospitalizations. Furthermore, patients with four or more chronic diseases showed the largest increase in health care cost, reaching CNY 3369.9 (*p* < 0.01) (Table 2).

### 3.3. Productivity Loss Estimation of Depressive Symptoms

**Figure 2:**
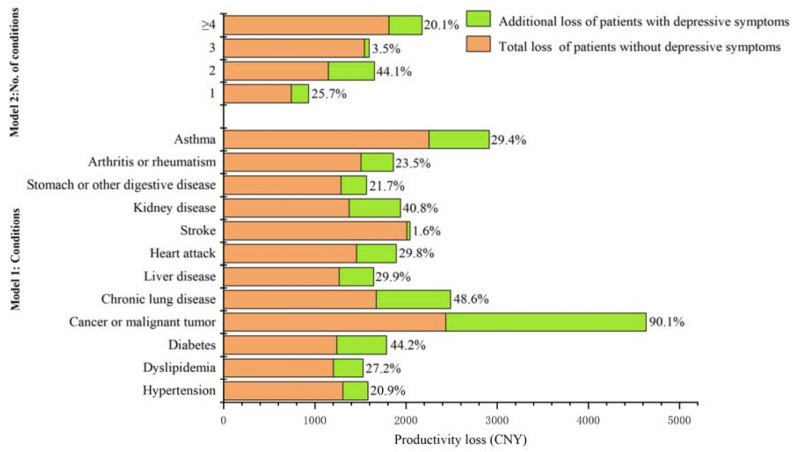
Comparing the proportion of additional productivity loss to the total loss of patients without depressive symptoms. Note: The data details used in drawing Figure 2 are shown in Table A3.

The presence of depressive symptoms was also associated with increased productivity loss among middle-aged and elderly people with chronic conditions. The amount of productivity loss associated with depressive symptoms accounted for 1.6% to 90.1% of productivity loss of patients without depressive symptoms among the 12 types of chronic disease groups (Figure 2). It was highest in patients with cancer or malignant tumors, which resulted in a loss of CNY 2196.2 (*p* < 0.05), followed by chronic lung disease and kidney disease, with an annual productivity loss of CNY 813.5 and CNY 561.7, respectively (*p* < 0.05 for both). The amount of productivity loss of patients with two chronic diseases was nearly 2.7 times higher than those with one chronic disease (CNY 506.1 vs. CNY 190.5, *p* < 0.05 for both). The productivity loss of patients with chronic conditions was mostly manifested in agricultural loss. Patients with kidney diseases and arthritis or rheumatism had the highest number of agricultural missed work days, at 6.1 and 6.0, respectively (*p* < 0.01 for both) (Table 3).

## 4. Discussion

Our study results showed that the prevalence rate of depressive symptoms in 12 types of chronic diseases groups was mostly higher than the existing research findings [13,37]. It might be due to the different measurement tools and different severity of disease. Consistent with previous studies, our results also showed that the incidence of depressive symptoms increased with the number of comorbidities [10]. 

Our study revealed that depressive symptoms were associated with greater health care costs in middle-aged and elderly people with chronic conditions. Co-occurring depressive symptoms could directly increase the economic burden of people with chronic conditions through psychotherapeutic, healthcare services and the use of antidepressants [38]. Patients with depressive symptoms in 12 common chronic diseases consistently showed marked increased outpatient visits and hospitalization costs. Patients may seek medical care for their depressive symptoms, thus substantially increasing their health care utilization, especially for outpatient services [39]. In addition, patients with depressive symptoms were usually unable to receive a timely diagnosis or effective treatment. It may exacerbate the deterioration of their physical health, leading to hospitalization, and increase the corresponding expenditure [40]. Previous studies have highlighted that mental health disorders were associated with increased health care utilization and expenditures, predominantly within one specific chronic disease [17,41]. However, our study highlighted different results and suggested that increased health care costs were associated with depressive symptoms in 12 common kinds of chronic disease. The results showed that depressive symptoms increased direct medical costs by 3.1% to 85.0% and the patients with or malignant tumors were most affected by depressive symptoms. A population-based cohort study in Canada showed that mental health disorders increased the medical costs of people with chronic diseases by 71.7%, which was higher than our results [18]. This is possibly due to the fact that our study population included the elderly, who report greater medical services utilization. In addition, the results did not clearly show that the health care cost associated with depressive symptoms was positively correlated with the number of chronic diseases. A study of patients with diabetes and hypertension found that depression was associated with higher health care costs [31]. This suggests that the effect of depressive symptoms on healthcare cost may vary by the type of comorbidity.

Our results showed that depressive symptoms were associated with increased productivity loss among people with chronic diseases. The economic loss of productivity was also highest in patients with cancer or malignant tumors, and it has grown by 90.14% based on an average annual economic loss of CNY 2436.6 in the corresponding non-depressed groups. The results suggested that depressive symptoms have limited the ability of people with chronic diseases to work and caused productivity loss, mainly reflected in agricultural labor. Consistent with a previous study based on the middle-aged and elderly Chinese population, depressive symptoms had a significant negative impact on their labor participation and working hours, especially for those living in rural areas [35]. Compared with urban residents, rural residents have a higher incidence of depressive symptoms, but they were less likely to receive adequate and timely treatment, thus compromising their ability to work [42]. Additionally, our results showed that employed people with chronic diseases had a higher risk of depressive symptoms, but we did not observe a higher economic loss of productivity associated with depressive symptoms in that population. This may be because employees did not immediately ask for leave even if they felt unwell [43]. As for multimorbidity conditions, we found that the effect of depressive symptoms on productivity loss did not increase as the number of chronic conditions increased. This could be due to the fact that the group with a larger number of comorbidities tended to be older and retired [20]. In our study, the indirect economic burden only included the loss of ability to work, and excluded the loss of household productivity, so retirees were considered to have no productivity loss. 

Given the significant impact on the economic burden of depressive symptoms, particularly in people with cancer or malignant tumors, implementing specific population screening and monitoring is critical. Patients rarely recognize depression as a separate disease because of the overlapping physical symptoms between chronic diseases and depression [44]. Therefore, screening for depressive symptoms can help patients receive proper treatment after diagnosis and prevent their symptoms from worsening, eventually reducing long-term medical costs [45]. More importantly, our results indicated that there was an urgent need to develop effective care models for patients with chronic diseases. Current chronic disease management measures are insufficient and have largely ignored the impact of mental health [46]. The integrated medical and psychiatric health care conducted by medical teams has been proven to be effective in improving health outcomes for chronic patients with depression [47]. In the integrated health setting, patients can continuously access a full cycle of health care services provided by a collaborative team [48]. The team generally consists of general practitioners, specialists, nurses, etc., and can provide comprehensive health care services, including disease treatment, management, monitoring and health education and social support [49]. Our result indicates that depressive symptoms mainly increased the need for primary care in chronic patients, such as outpatient consultation and medication. So, public health systems should pay attention to raising awareness and improving clinical knowledge of depression in primary care practitioners and implement primary care-based collaborative and integrated care.

Our study demonstrated that depressive symptoms affect work productivity of people with chronic diseases. Although the increased amount was not large, the productivity loss in the active labor force group would be even higher among the working-age population [50]. Thus, the government should strengthen labor security for people with chronic diseases and reduce their restrictions on labor compensation. Workplace health promotion (WHP) strategies should be considered, with a particular focus on the prevention of mental health conditions. In many countries, WHP strategies have been proven to be effective in maintaining and enhancing productivity by preventing, minimizing and eliminating health hazards [51]. The efforts of mental-health-based WHP strategies among people with chronic diseases could focus on encouraging screening for depression and reducing anxiety or depression risks, such as limiting smoking and alcohol abuse. In addition, individuals may be afraid to disclose their depression and reluctant to take time off because they might lose their jobs, and even though they may remain at work, they have lower levels of productivity [43]. Consequently, before implementing prevention programs and treating mental illness at the workplace, it is important to address the underlying problems associated with stigma and support [52].

This study was subject to several limitations. First, the study employed a cross-sectional design, which limited our understanding of causality. Population-based cohort studies are needed in further research. Second, our study used retrospectively collected data, and recorded a range value for the uncertain health care cost, which may generate information bias. Third, we did not acquire data on lost household productivity and the productivity loss caused by caring for sick family members, which may underestimate the indirect economic burden, especially for the old population. Fourth, when measuring the productivity loss, we considered the retired population to have no productivity loss, which may lead to an underestimation of the productivity loss. Last, we did not have any information on the disease trajectory, and thus were not able to delineate whether severe physical chronic diseases led to the development of mental health issues, and if mental diseases then lead to higher health care costs through exacerbating the development of chronic physical disease.

## 5. Conclusions

Our study is the first to calculate the economic burden associated with depressive symptoms among patients with 12 common chronic diseases. In summary, depressive symptoms increased health care costs and productivity loss among people with chronic diseases, particularly for patients with cancer or malignant tumors. However, the increased economic burden was not found to increase with the number of physical chronic diseases. The excess economic burden suggests that government and health care systems should give priority to mental health issues of people with chronic diseases. Future research should focus on assessing the effectiveness of psychological interventions in people with chronic diseases and comparing the cost-effectiveness of different intervention models.

## Figures and Tables

**Table 1:** Demographic characteristics of those with depressive symptoms and without depressive symptoms among chronic patients.

Character Variables	With Depressive Symptoms	Without Depressive Symptoms	*p*-Value
	*n* = 4959 (%)	*n* = 7499 (%)	
Gender			
Male	1896 (38.2)	4064 (54.2)	<0.001
Female	3063 (61.8)	3435 (45.8)	
Age			
45–54	1124 (22.7)	1816 (24.2)	0.175
55–64	1759 (35.5)	2604 (34.7)	
65–74	1485 (30.0)	2241 (30.0)	
≥75	591 (11.9)	838 (11.2)	
Marital status			
Married	4116 (83.0)	6667 (88.9)	<0.001
Widowed	104 (2.1)	132 (1.8)	
Separated/Divorced/Unmarried	739 (14.9)	700 (9.3)	
Residence district			
Rural	4161 (83.91)	5399 (72.0)	<0.001
Urban	798 (16.09)	2100 (28.0)	
Education level			
No formal education	2571 (51.8)	2678 (35.7)	<0.001
Primary education	1130 (22.8)	1745 (23.3)	
Secondary education	881 (17.8)	1883 (25.1)	
High school and above	377 (7.6)	1193 (15.9)	
Occupation			
Retired/Receded	299 (6.0)	859 (11.5)	<0.001
Unemployed	1611 (32.5)	2042 (27.2)	
Agricultural work	386 (7.8)	916 (12.2)	
Employed	2354 (47.5)	3092 (41.2)	
Self-employed/Unpaid helper	309 (6.2)	590 (7.9)	
Medical insurance			
Urban employee medical insurance	420 (8.5)	1408 (18.8)	<0.001
New cooperative medical insurance	3561 (71.8)	4456 (58.4)	
Urban and rural resident medical insurance	571 (11.5)	949 (12.7)	
Urban resident medical insurance and others	248 (5.0)	527 (7.0)	
No insurance	159 (3.2)	159 (2.1)	
Household monthly expenditure			.
Lowest 25%	1562 (31.5)	1647 (22.0)	<0.001
Lower 25%	1573 (31.7)	2266 (30.2)	
Higher 25%	875 (17.6)	1520 (20.3)	
Highest 25%	949 (19.1)	2066 (27.6)	
Perceived health status			
Good	498 (10.0)	1944 (25.9)	<0.001
Fair	2223 (44.8)	4183 (55.8)	
Poor	2238 (45.1)	1372 (18.3)	
No. of chronic disease			
1	1295 (26.1)	2878 (38.4)	<0.001
2	1277 (25.8)	2069 (27.6)	
3	980 (19.7)	1278 (17.0)	
≥4	1407 (28.4)	1274 (17.0)	

**Table 2:** Additional health care use and cost among chronic patients with depressive symptoms compared with chronic patients without depressive symptoms.

Condition	Total Health Care Cost (95% CI)	Outpatient		Hospitalization		Self-Medication	
		Visit (95% CI)	Cost (95% CI)	Visit (95% CI)	Cost (95% CI)	Visit (95% CI)	Cost (95% CI)
Model 1: conditions							
Hypertension	2371.8 (933.8–3809.9) **	1.4 (0.6–2.2) **	872.1 (1.1–1745.2) *	0.05 (0.02–0.1) **	1191.4 (440.8–1942.1) **	0.7 (0.4–1.1) **	476.7 (150.4–803.0) **
Dyslipidemia	3009.9 (1134.4–4885.3) **	1.8 (0.8–2.8) **	379.5 (−738.1–1497.1)	0.1 (0.04–0.2) **	1155.7 (304.8–2006.6) **	0.6 (0.2–1.1) **	702.0 (271.6–1132.4) **
Diabetes	295.1 (−1653.9–2244.1)	1.3 (−0.01–2.36)	−843.2 (−1916.7–230.3)	0.05 (−0.03–0.13)	−174.0 (−1112.9–746.8)	0.4 (−0.1–1.0)	549.1 (48.1–1049.9) *
Cancer or malignant tumor	17,273.7 (6164.7–28,382.6) **	2.6 (−0.1–5.3)	1765.0 (−21,986.7–57,286.6)	0.5 (0.21–0.84) **	19,158.6 (7180.2–31,136.9) **	2.3 (0.8–3.8) **	555.9 (−695.0–1806.8)
Chronic lung disease	4759.4 (2321.8–7197.0) **	2.0 (0.8–3.3) **	1707.3 (295.2–3119.5) *	0.1 (0.03–0.18) *	1616.01 (601.9–2630.1) **	0.9 (0.4–1.4) **	637.4 (287.3–987.5) **
Liver disease	1611.2 (−1017.6–4234.0)	1.3 (−0.5–3.1)	65.2 (−1183.0–1313.4)	0.04 (−0.11–0.19)	−27.7 (−1062.4–1007.1)	0.8 (0.01–1.6) *	519.3 (−145.7–1184.4)
Heart attack	2359.5 (234.41–4484.6) *	1.4 (0.4–2.4) **	425.2 (−600.5–1450.9)	0.1 (0.02–0.16) **	859.0 (−265.2–1983.2)	0.5 (0.1–0.9) *	734.2 (239.1–1229.3) **
Stroke	2595.0 (−488.7–5678.7)	0.2 (−2.0–2.3)	957.3 (33.7–1880.94) *	0.3 (−0.07–0.62)	1631.4 (64.5–3198.2) *	0.1 (−0.7–0.9)	123.9 (−496.2–744.0)
Kidney disease	1178.4 (−1423.5–3780.3)	2.2 (0.6–3.7) **	672.3 (−410.8–1755.4)	0.04 (−0.58–0.67)	494.9 (−1063.6–2053.5)	0.5 (−0.2–1.1)	−59.1 (−683.2–565.0)
Stomach or other digestive disease	1334.4 (−61.5–2730.36)	1.8 (0.9–2.7) **	287.2 (−631.4–1205.8)	0.05 (0.01–0.88) *	527.8 (−1.7–1057.4) *	0.8 (0.4–1.1) **	494.6 (210.0–779.3) **
Arthritis or rheumatism	1909.3 (429.8–3388.9) *	1.1 (0.3–1.9) **	764.1 (−213.4–1741.5)	0.05 (0.01–0.09) **	279.5 (−171.7–730.6)	0.6 (0.1–1.1) *	624.0 (348.0–900.1) **
Asthma	3589.0 (640.5–6537.4) *	1.1 (−0.7–3.0)	2353.4 (474.3–4232.6) *	0.1 (−0.02–0.30)	−331.4 (−1724.1–1062.4)	0.5 (−0.3–1.2)	740.8 (195.0–1286.6) **
Model 2: No. of conditions							
1	2422.7 (756.6–4088.8) **	0.8 (0.1–1.4) *	1720.8 (507.9–2933.6) **	0.1 (0.01–0.13) *	373.9 (28.8–718.9) *	0.9 (0.5–1.31) **	113.0 (−150.2–376.2)
2	1365.7 (3.8–2727.5) *	0.9 (0.03–1.7) *	820.1 (−109.5–1749.6)	0.01 (−0.04–0.05)	173.7 (−319.8–667.3)	0.7 (0.3–1.1) **	459.1 (134.0–784.2) **
3	1674.4 (−4.4–3353.3)	2.0 (0.7–3.2) **	624.7 (−351.0–1600.3)	0.1 (0.01–0.13) *	928.1 (−3.5–1859.7) *	0.6 (0.1–1.1) *	395.5 (−31.8–822.9)
≥4	3369.9 (924.3–5815.5) **	1.6 (0.3–2.9) *	444.4 (−968.4–1856.9)	0.1 (0.04–0.2) **	999.7 (−95.2–2094.7)	0.5 (0.1–0.9) *	849.6 (334.8–1364.3) **

Note: (1) All estimated results using robust standard errors and multivariable regression adjusted by gender, age, marital status, residence district, education level, perceived health status and comorbidity status. (2) * *p* < 0.05, ** *p* < 0.01.

**Table 3:** Additional missed days and productivity loss among chronic patients with depressive symptoms compared with chronic patients without depressive symptoms.

Condition	Total Productivity Loss (95% CI)	Unemployment		Agricultural		Employed	
		Missed Days (95% CI)	Economic Loss (95% CI)	Missed Days (95% CI)	Economic Loss (95% CI)	Missed Days (95% CI)	Economic Loss (95% CI)
Model 1: conditions							
Hypertension	273.5 (16.8–530.2) *	1.9 (−0.9–4.6)	22.3 (−218.5–263.1)	3.5 (2.1–4.9) **	164.6 (79.7–249.5) **	0.01 (−0.01–0.3)	0.03 (−0.1–0.2)
Dyslipidemia	326.8 (23.8–629.7) *	3.6 (0.4–6.9) *	76.7 (−208.01–361.4)	2.7 (1.2–4.2) **	151.8 (66.8–236.8) **	0.01 (−0.01–0.02)	0.8 (0.1–1.5) *
Diabetes	547.3 (112.9–981.7) *	2.3 (−1.6–6.2)	1.9 (−0.7–4.5)	3.6 (1.4–5.8) **	100.0 (4.1–196.0) *	0.02 (−0.01–0.04)	0.1 (−0.4–0.6)
Cancer or malignant tumor	2196.2 (244.0–4149.0) *	4.5 (−1.2–8.0)	1195.7 (−804.5–3195.9)	3.8 (2.3–5.3) **	137.5 (3.0–171.9) *	0.1 (−0.1–0.2)	−3.4 (−9.8–3.5)
Chronic lung disease	813.5 (330.7–1296.3) **	2.1 (−3.0–7.2)	451.2 (4.0–898.4) *	5.3 (2.8–7.8) **	286.8 (135.2–438.3) **	0.01 (−0.01–0.03)	−0.01 (−0.1–0.5)
Liver disease	378.7 (−108.14–865.4)	1.8 (−4.7–9.1)	6.2 (−9.0–14.5)	4.4 (1.4–7.5) **	93.5 (−35.3–222.2)	0.03 (−0.03–0.1)	0.3 (0.03–0.6) *
Heart attack	434.5 (11.5–857.5) *	2.5 (−1.6–6.7)	49.2 (−304.1–402.4)	4.0 (2.1–5.9) **	224.2 (114.1-−334.4) **	0.04 (−0.5–0.6)	0.02 (−0.05–0.1)
Stroke	32.6 (−351.2–405.9)	3.2 (−6.4–12.9)	63.5 (−514.3–767.3)	5.3 (2.7–7.9) **	129.0 (57.2–200.9) **	0.4 (−0.3–1.1)	0.03 (0.01–0.15) *
Kidney disease	561.7 (56.6–1066.9) *	1.8 (−2.1–7.3)	279.4 (−169.4–728.1)	6.1 (3.1–9.1) **	163.0 (24.5–301.6) *	0.3 (−0.01–0.5)	33.8 (11.6–55.9) **
Stomach or other digestive disease	278.9 (38.1–519.7) *	1.5 (0.29–3.3) *	168.4 (−61.6–398.3)	3.7 (2.1–5.3) **	167.5 (77.6–257.4) **	0.2 (−0.1–0.5)	35.4 (2.79–68.1) *
Arthritis or rheumatism	354.4 (115.1–593.6) **	1.1 (−1.8–3.9)	−44.1 (−276.4–188.1)	6.0 (4.3–7.7) **	306.7 (211.5–402.0) **	0.2 (−0.04–0.3)	17.1 (−4.0–38.2)
Asthma	661.8 (−314.5–1638.2)	1.3 (−1.1–4.5)	−56.1 (−290.4–178.2)	2.6 (−2.3–7.4)	139.8 (−60.4–339.9)	−0.1 (−0.9–0.7)	−0.02 (−0.05–0.03)
Model 2: No. of conditions							
1	190.5 (17.0–364.1) *	1.0 (−0.7–2.7)	128.6 (−9.0–266.3)	0.8 (0.07–1.45) *	118.7 (46.7–190.7) **	0.1 (−0.2–0.4)	24.1 (−9.4–57.5)
2	506.1 (193.7–818.5) **	2.8 (−0.7–6.3)	110.7 (−162.0–383.3)	0.9 (0.03–1.70) *	118.4 (48.0–188.9) **	0.01 (−0.3–0.4)	13.2 (−10.2–36.6)
3	54.0 (−338.2–446.2)	0.5 (−3.6–4.7)	−166.9 (−561.4–227.6)	2.0 (0.67–3.21) **	249.2 (125.2–373.3) **	0.05 (−0.2–0.3)	−0.01 (−0.03–0.02)
≥4	363.4 (−70.6–797.4)	3.0 (−1.8–7.9)	47.7 (−357.7–453.1)	1.6 (0.30–2.85) *	119.9 (49.3–350.6) **	0.05 (−0.2–0.3)	0.3 (−0.2–0.8)

Note: (1) All estimated results using robust standard errors and multivariable regression adjusted by gender, age, marital status, residence district, education level, perceived health status and comorbidity status. (2) * *p* < 0.05, ** *p* < 0.01.

## Data Availability

The datasets generated and/or analyzed during the current study are available in the CHARLS repository, at http://charls.pku.edu.cn/index/en.html, accessed on 23 December 2021.

## Appendix A

**Table A1:** Characteristics of chronic patients and prevalence of depressive symptoms from CHARLS survey in 2018 (*n* = 12,458).

Conditions ^a^	Chronic Patients *n* (%) ^b^	Present Depressive Symptoms *n* (%) ^c^
Model 1: conditions		
Hypertension	5773 (46.3)	2294 (39.7)
Dyslipidemia	3276 (26.3)	1303 (39.8)
Diabetes	1901 (15.3)	789 (41.5)
Cancer or malignant tumor	297 (2.4)	137 (46.1)
Chronic lung disease	2220 (17.8)	1054 (47.5)
Liver disease	982 (7.9)	440 (44.8)
Heart attack	2831 (22.7)	1289 (45.5)
Stroke	885 (7.1)	431 (48.7)
Kidney disease	1383 (11.1)	683 (49.4)
Stomach or other digestive disease	4553 (36.6)	2177 (47.8)
Arthritis or rheumatism	5790 (46.5)	2779 (48.0)
Asthma	801 (6.4)	416 (51.9)
Model 2: No. of conditions ^d^		
1	4173 (33.5)	1295 (31.0)
2	3346 (26.9)	1277 (38.2)
3	2258 (18.1)	980 (43.4)
≥4	2681 (21.5)	1407 (52.5)

Note: (1) a: Individual chronic diseases conditions were not mutually exclusive. (2) b: The proportion of each chronic disease in the total study sample. (3) c: The proportion of patients who presented depressive symptoms for each chronic disease. (4) d: Total co-morbidities included the listed chronic physical conditions.

**Table A2:** Comparing the proportion of health care costs associated with depressive symptoms to total costs of patients without depressive symptoms in 12 common chronic diseases.

Conditions	Without Depressive Symptoms	With Depressive Symptoms	%
	Total Cost (95% CI)	Additional Cost (95% CI)	
Model 1: conditions			
Hypertension	7750.5 (6762.4–8738.5)	2371.8 (933.8–3809.9)	30.6
Dyslipidemia	8898.1 (7612.8–10,183.4)	3009.9 (1134.4–4885.3)	33.8
Diabetes	9674.5 (8115.1–11,234.0)	2,95.1 (−1653.9–2244.1)	3.1
Cancer or malignant tumor	20,318.6 (13,443.5–27,193.8)	17,273.7 (6164.7–28,382.6)	85.0
Chronic lung disease	9010.2 (7283.6–10,736.8)	4759.4 (2321.8–7197.0)	52.8
Liver disease	8870.5 (7067.5–10,673.4)	1611.2 (−1017.6–4234.0)	18.2
Heart attack	11,413.0 (9657.6–13,168.5)	2359.5 (234.4–4484.6)	20.7
Stroke	11,609.6 (8357.6–14,861.6)	2595.0 (−488.7–5678.7)	22.4
Kidney disease	10,937.1 (8925.5–12,948.7)	1178.4 (−1423.5–3780.3)	10.8
Stomach or other digestive disease	7893.1 (6567.2–9219.0)	1334.4 (−61.5–2730.4)	16.9
Arthritis or rheumatism	7399.1 (6382.3–8415.9)	1909.3 (429.8–3388.9)	25.8
Asthma	10,232.4 (7709.6–12,755.2)	3589.0 (640.5–6537.4)	35.1
Model 2: No. of conditions			
1	3764.9 (3152.3–4377.4)	2422.7 (756.6–4088.8)	64.4
2	6363.7 (5405.7–7321.6)	1365.7 (3.8–2727.5)	21.5
3	7932.1 (6315.6–9550.1)	1674.4 (−4.4–3353.3)	21.1
≥4	12,570.8 (10,594.1–14,547.5)	3369.9 (924.3–5815.5)	26.8

**Table A3:** Comparing the proportion of productivity loss associated with depressive symptoms to total productivity loss of patients without depressive symptoms in 12 common chronic diseases.

Conditions	Without Depressive Symptoms	With Depressive Symptoms	%
	Total Loss (95% CI)	Additional Loss (95% CI)	
Model 1: conditions			
Hypertension	1306.7 (1167.3–1446.1)	273.5 (16.8–530.2)	20.9
Dyslipidemia	1203.3 (1025.2–1381.3)	326.8 (23.8–629.7)	27.2
Diabetes	1239.7 (991.7–1487.7)	547.3 (112.9–981.7)	44.2
Cancer or malignant tumor	2436.6 (1524.2–3349.0)	2196.2 (244.0–4149.0)	90.2
Chronic lung disease	1673.5 (1406.7–1940.2)	813.5 (330.7–1296.3)	48.6
Liver disease	1266.0 (932.7–1599.2)	378.7 (−108.14–865.4)	29.9
Heart attack	1458.8 (1236.3–1681.2)	434.5 (11.5–857.5)	29.8
Stroke	2009.8 (1535.6–2484.0)	−22.6 (−851.2–805.9)	1.6
Kidney disease	1376.5 (1068.4–1684.5)	561.7 (56.6–1066.9)	40.8
Stomach or other digestive disease	1288.2 (1128.6–1447.8)	278.9 (38.1–519.7)	21.7
Arthritis or rheumatism	1507.2 (1352.6–1661.9)	354.4 (115.1–593.6)	23.5
Asthma	2251.8 (1717.0–2786.7)	661.8 (−314.5–1638.2)	29.4
Model 2: No. of conditions			
1	742.5 (632.0–853.0)	190.5 (17.0–364.1)	25.7
2	1147.8 (983.9–1311.6)	506.1 (193.7–818.5)	44.1
3	1543.2 (1290.1–1796.3)	54.0 (−338.2–446.2)	3.5
≥4	1812.3 (1547.6–2076.9)	363.4 (−70.6–797.4)	20.1
